# Supplementary material for: Enhanced IR Radiative Cooling of Silver Coated PA Textile
Source: Polymers (Basel). 2021 Dec 31;14(1):147. doi: 10.3390/polym14010147 (PMC8747296; doi:10.3390/polym14010147)
Supplement: Supplementary file 1 [file polymers-14-00147-s001.zip › polymers-1507543-SI.pdf]

Supplementary Materials for

Enhanced IR radiative cooling of silver coated PA textile

Xiaoyu Xie, Yang Liu, Ying Zhu, Zhao Xu, Yanping Liu, Dengteng Ge, Lili Yang*

*Corresponding author: Lili Yang (liliyang@dhu.edu.cn)

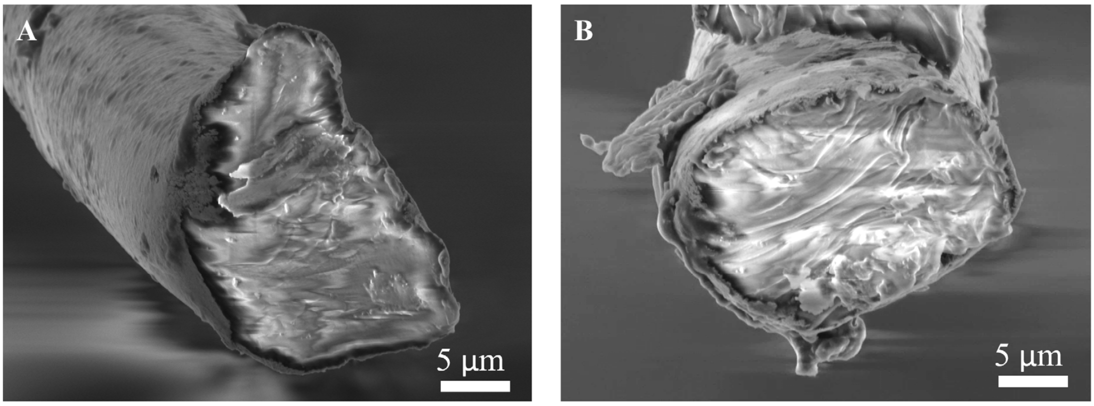

Figure. S1 Cross-sectional SEM images of Ag@PA fiber.

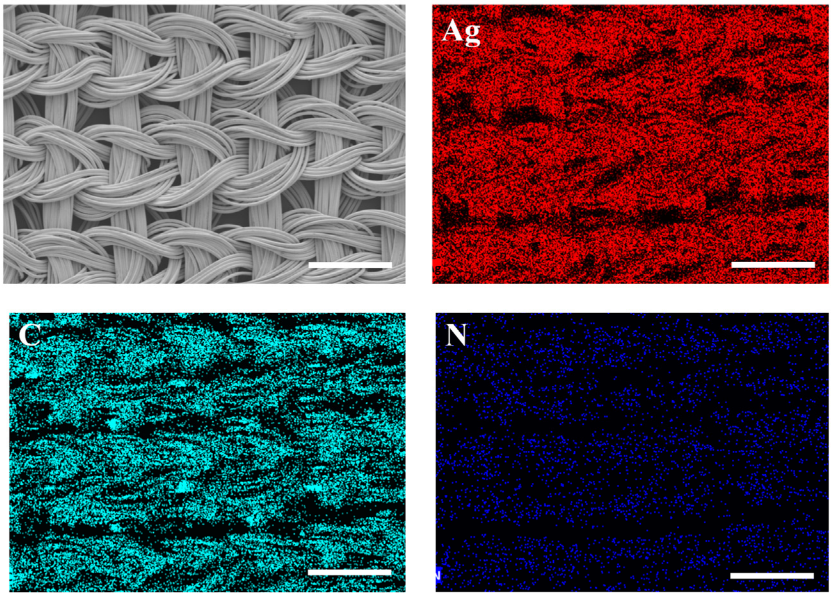

Figure. S2 SEM and EDS images of Ag@PA fabric. Scale bar: 500 μm.

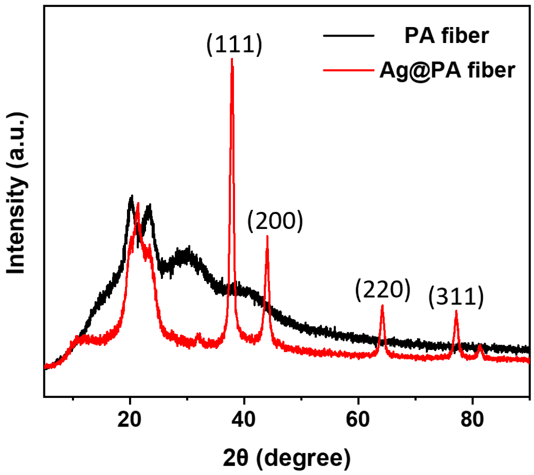

Figure. S3 XRD patterns of PA fiber and Ag@PA fiber

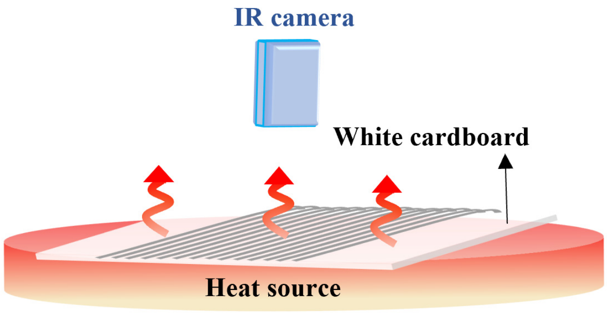

Figure. S4 Schematic diagram for fiber bundle emissivity measurement

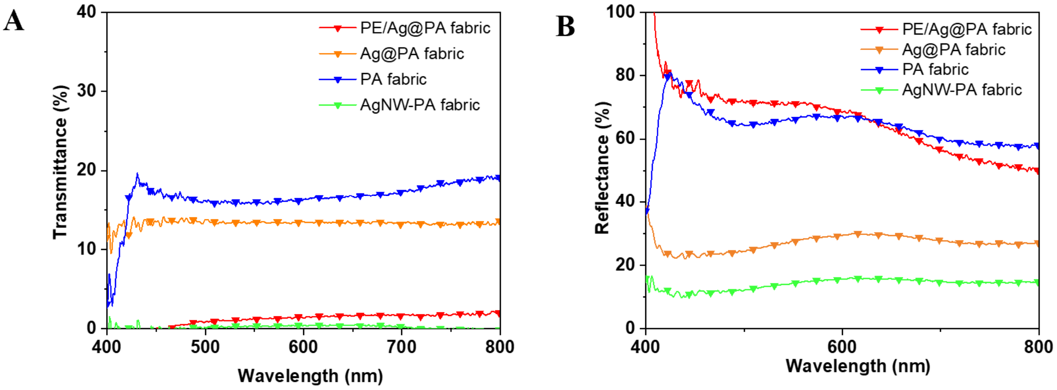

Figure. S5 Visible transmittance and reflection of different fabrics
